# Clinical Efficacy of Probiotics for Relieving Cold Symptoms in Healthy Individuals: A Randomized, Double-Blind, Placebo-Controlled Clinical Trial

**DOI:** 10.3390/nu17091490

**Published:** 2025-04-28

**Authors:** Lisa Lungaro, Patrizia Malfa, Francesca Manza, Matilde Negrelli, Anna Costanzini, Diletta Francesca Squarzanti, Marta Lo Re, Alessio Cariani, Sara Ghisellini, Fabio Caputo, Alfredo De Giorgi, Pasquale Mansueto, Antonio Carroccio, Roberto De Giorgio, Giacomo Caio

**Affiliations:** 1 Department of Translational Medicine, University of Ferrara, 44121 Ferrara, Italy; francesca.manza@unife.it (F.M.); matilde.negrelli@edu.unife.it (M.N.); anna.costanzini@unife.it (A.C.); fabio.caputo@unife.it (F.C.); alfredo.degiorgi@unife.it (A.D.G.); roberto.degiorgio@unife.it (R.D.G.); 2 Geriatric Unit, P. Giaccone University Hospital, 90127 Palermo, Italy; pasquale.mansueto@policlinico.pa.it; 3 SynBalance srl, 21040 Origgio, Italy; p.malfa@synbalance.care (P.M.); d.squarzanti@synbalance.care (D.F.S.);; 4 Academic Unit of Gastroenterology, Sheffield Teaching Hospitals NHS Foundation Trust, Sheffield S5 7AT, UK; 5 Clinical Pathology Unit, S. Anna University Hospital, 44124 Ferrara, Italy; a.cariani@ospfe.it (A.C.); s.ghisellini@ospfe.it (S.G.); 6 Unit of Internal Medicine, “V. Cervello” Hospital, Ospedali Riuniti “Villa Sofia-Cervello”, 90146 Palermo, Italy; antonio.carroccio@unipa.it; 7 Department of Health Promotion Sciences, Maternal and Infant Care, Internal Medicine and Medical Specialties (PROMISE), University of Palermo, 90127 Palermo, Italy; 8 Internal Medicine Unit, S.S. Annunziata Hospital, Cento, 44042 Ferrara, Italy; 9 Mucosal Immunology and Biology Research Center, Massachusetts General Hospital-Harvard Medical School, Boston, MA 02114, USA; 10 Celiac Disease and Allergology Center, St. Anna University Hospital, 44124 Ferrara, Italy

**Keywords:** probiotics, cold symptoms, food supplements, immunity, URTIs

## Abstract

**Background:** Colds are widespread infectious diseases that affect daily life, increasing healthcare costs and limiting productivity. **Objectives**: The aim of this study was to investigate the effects of a dietary supplement containing specific probiotic strains (*L. plantarum* PBS067, *L. acidophilus* PBS066, *B. lactis* BL050) on cold symptom relief, immune response enhancement, and quality of life. **Methods** This randomized, double-blind, placebo-controlled trial included 65 healthy volunteers (age range: 18–44 years), divided into two groups: 40 received the probiotic treatment (with vitamins and bulking agents), and 25 received placebo (vitamins and bulking agents only) for 12 weeks. Cold symptoms and systemic inflammation were assessed at three time points (baseline T0, post-treatment T1, and 6 weeks after treatment T2). **Results:** Probiotics were associated with a shorter average duration of cold symptoms (4.5 *vs.* 6.7% for Placebo, *p* < 0.05). At T1, fever and muscle pain occurred in 20% of participants in the Probiotic group *vs.* 28% and 44% in the Placebo group, respectively (*p* < 0.05 for muscle pain *vs.* Placebo). For muscle pain, a trend was maintained also at T2 (17.5% *vs.* 20%). The pro-inflammatory cytokine IFN-γ levels significantly decreased in the Probiotic group *vs.* T0 (*p* < 0.0001 at T1 and *p* < 0.01 at T2), while they increased in the Placebo group (22.279 ± 3.538 *vs.* 19.432 ± 3.143 pg/mL, *p* = NS). Although not statistically significant, at T1 the Probiotic group had higher levels of IL-10 *vs*. T0 (266.98 ± 78.432 *vs.* 240.967 ± 70.238, pg/mL *p* = NS). **Conclusions:** The probiotic mix effectively alleviated cold symptoms and reduced pro-inflammatory cytokine levels, suggesting anti-inflammatory effects.

## 1. Introduction

The term “cold” refers to various respiratory illnesses common in colder seasons, primarily caused by highly contagious viral infections [1]. Cold symptoms typically manifest two to three days post-infection, including nasal congestion, excessive mucus, sinus inflammation, sneezing, sore throat, cough, and headache [2,3]. Recovery generally takes about a week but can extend to two weeks. In adults, cold usually presents mild symptoms affecting the upper respiratory system, like sore throat and runny nose. However, in children and immunocompromised patients, colds can lead to lower respiratory infections and potentially serious complications (i.e., bronchitis and pneumonia) [3]. Colds are the most prevalent infectious disease globally, often resulting from viral agents, with over two hundred strains identified. Rhinoviruses account for 30–35% of colds in adults, peaking in autumn, spring, and summer, while coronaviruses are more common in winter and early spring [4]. Other viruses (e.g., adenoviruses and enteroviruses) also contribute to colds. Specific viral agents can cause more serious conditions, such as those linked to Respiratory Syncytial Virus (RSV) and parainfluenza virus, potentially resulting in bronchial and pulmonary diseases [5]. Cold-related illnesses significantly impact daily activities and overall quality of life (QoL), threatening physical and mental health [2]. They also contribute to social challenges, such as increased healthcare costs, reduced productivity, and economic losses from absenteeism.

The gut hosts over 70% of immune cells and its connections with the intestinal microbiota are crucial for the development of innate and adaptive immunity and to maintain immune tolerance [6]. Research into the gut microbiota has revealed its complex interactions with the central nervous, endocrine, and immune systems [7]. Intestinal microorganisms perform essential physiological functions, aiding the bio-conversion of food components and producing various bioactive molecules (e.g., short-chain fatty acids, vitamins, and neurotransmitters). These molecules enhance immune and endocrine responses and offer protection against colonization of pathogens. The gut microbiota also plays a crucial role in maintaining metabolic and immunological homeostasis, significantly affecting human physiology. Among its contributions, short-chain fatty acids (SCFAs), such as acetic, butyric, and propionic acids, are particularly noteworthy for their role in stimulating the immune system and maintaining intestinal barrier function [8]. Dysbiosis, which is an imbalance in the intestinal microbiota, can contribute to intestinal mucosa tight junction dysfunction and increased permeability. These mechanisms trigger inflammatory responses, which result in the production of pro-inflammatory cytokines.

In this context, probiotics—live microorganisms that provide health benefits when consumed adequately—are proposed as a supportive measure for alleviating symptoms associated with cold-related illnesses. Evidence indicates that probiotics can help restore the balance of the gut microbiome and synthesize bacteriocins, which inhibit harmful pathogens [9]. Bacteriocins exhibit significant immunomodulatory effects, promote anti-inflammatory and inhibit pro-inflammatory cytokines. Moreover, probiotics promote mucin production, which helps strengthen the integrity of the intestinal barrier [10] and prevents pathogens from entering the bloodstream, with a reduced likelihood of systemic infections. Extensive research explored the effectiveness of probiotics, particularly for gastrointestinal disorders and bacterial infections across oral, respiratory, intestinal, and urogenital systems. A growing number of studies examined the role of probiotics in treating respiratory infections and cold symptoms. Orally-supplemented probiotics are recognized for enhancing innate immunity and elevating antibody levels against enteric infections caused by bacteria or viruses [11,12,13]. Previous data showed that probiotics could reduce the occurrence of upper respiratory tract infections (URTIs) or the common cold in high-risk groups, including children, elderly people [14,15,16], and elite athletes [17]. However, the specific molecular dynamics of how intestinal mucosal responses to probiotic species remain to be thoroughly explored. Despite this, the immunoregulatory effects of probiotics seem to be partly linked to their bacterial components, such as cell wall elements like peptidoglycan [18] and lipoteichoic acid [19], as well as internal bacterial components including DNA, RNA [20,21,22,23], and exopolysaccharides (EPS) [24].

In this context, specific probiotic strains— i.e., *Lactiplantibacillus plantarum* PBS067, *Lactobacillus acidophilus* PBS066, and *Bifidobacterium animalis* subsp*. lactis* BL050— exhibited encouraging results in vitro because of their antimicrobial properties, notable anti-inflammatory effects, ability to adhere to the intestinal epithelium, and antioxidant activity [9,25]. Furthermore, this probiotic composition was investigated in a clinical study to evaluate the effects on the immune system of elderly volunteers during winter. The improvement was highlighted by a reduction in the incidence of symptoms of common infectious diseases, the stimulation of the intestinal immune system, and the modulation of the respiratory tract immune system response [26].

This study aims to assess the clinical effectiveness of a food supplement that includes a combination of probiotic strains (*L. plantarum* PBS067, *L. acidophilus* PBS066, *B. lactis* BL050) in addressing cold symptoms in healthy individuals and to determine its impact on improving the immune response. This study will investigate the occurrence of common cold symptoms, the QoL, and the modulation of immunological parameters associated with inflammation.

## 2. Materials and Methods

**Figure 1:**
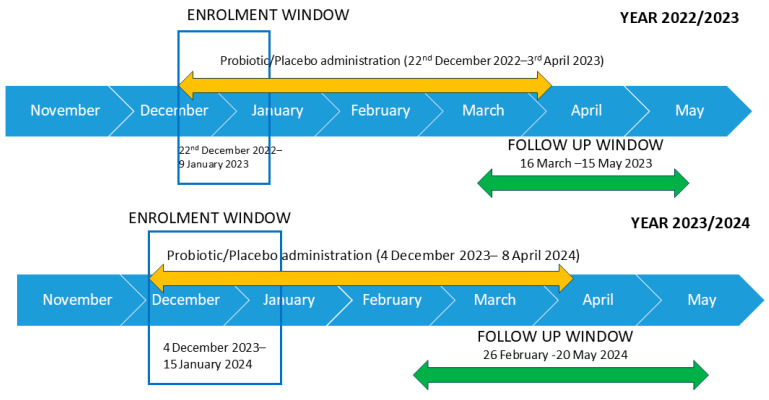
Study timeline. Seventy-five healthy volunteers aged 18–44 were recruited and screened during winter in the years 2022–2024 (16 subjects were recruited and screened in December 2022, 20 January 2023, 26 December 2023, 13 January 2024, respectively). Ten participants dropped out of the study (6 for personal reasons and 4 for antibiotic use).

This study is a randomized, double-blind, placebo-controlled, parallel-group clinical study (RDBPCT) conducted at St. Anna University Hospital, Cona (Ferrara), Italy, during the winter seasons between December 2022 and May 2024. With a 10% margin of error and 95% confidence level, a maximum of 65 patients would be required to represent the proportion of the Italian population aged 18–44 with a cold. Seventy-five healthy volunteers aged 18–44 were recruited and screened during winter (Figure 1). Of these, 10 patients dropped out of the study, 6 for personal reasons and 4 for antibiotic use (an exclusion criterion). Thus, the final number of enrolled participants was 65. Applying unequal randomization, patients were divided into a placebo (Placebo, n = 25) and a probiotic group (Probiotic, n = 40) [27]. This study was approved by the Ethics Committee Area Vasta Emilia Centro of the Emilia–Romagna Region (CE-AVEC) number 766/2021/Sper/AOUFe and registered on Clinical-Trial.gov (NCT05656729) on 5 December 2022. All the patients signed the informed consent. The inclusion and exclusion criteria for healthy participants of both genders are detailed in Supplementary Table S1.

### 2.1. Study Product

The probiotic formulation used in this study, including *Lactiplantibacillus plantarum* PBS067 (DSM 24937), *Lactobacillus acidophilus* PBS066 (DSM 24936), *Bifidobacterium animalis* subsp. *lactis* BL050 (DSM 25566), and the placebo product were provided by SynBalance Srl (Origgio, VA, Italy). The probiotic food supplement (Probiotic) was formulated in capsules of hydroxypropylmethylcellulose and pectin, containing *L. plantarum* PBS067 1 × 10^9^ CFU, *L. acidophilus* PBS066 1 × 10^9^ CFU, *B. lactis* BL050 1 × 10^9^ CFU plus vitamin B12, vitamin B6, folic acid, and bulking agents (maltodextrin, corn starch, and magnesium salt of fatty acids). The placebo was formulated in identical capsules and contained vitamin B12, vitamin B6, folic acid, and bulking agents only.

### 2.2. Study Design

**Figure 2:**
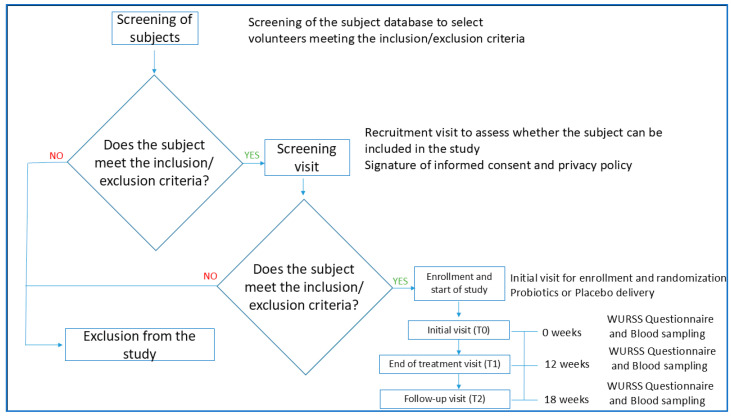
Flowchart of the study.

The probiotic group was treated with a combination of probiotic strains (*L. plantarum* PBS067, *L. acidophilus* PBS066, *B. lactis* BL050) along with vitamins B6 and B12, folic acid, and bulking agents normally found in food supplement formulations. The placebo group received a product containing vitamin B12, vitamin B6, folic acid, and bulking agents only. Participants who consented to participate in the study were instructed to refrain from using any other dietary supplements containing probiotics or consuming probiotic-rich foods, such as fermented products, yogurt, and probiotic-enriched food. Participants consumed one capsule daily, containing either the probiotic formulation or the placebo, taken orally away from meals for 12 weeks. The effects of the treatment were evaluated at baseline (T0), at the end of treatment (after 12 weeks, T1), and after 6 weeks from the end of treatment (T2, or follow-up). The total period of the study observation was 18 weeks. Cold symptoms and QoL were evaluated by a validated questionnaire (WURSS-21) at each time point. Systemic inflammation was assessed by cytokine dosage (i.e., interferon-gamma, IFN-γ, and interleukin-10, IL-10) at T0, T1, and T2. The study flowchart is shown in Figure 2.

### 2.3. Questionnaire

#### Wisconsin Upper Respiratory Symptom Survey (WURSS)

The Wisconsin Upper Respiratory Symptom Survey (WURSS) is a validated questionnaire to evaluate self-reported symptoms of cold. The WURSS-21 comprises 20 questions, 11 related to cold symptoms and 9 assessing the impact of cold on daily activities [28]. An additional question investigates whether the cold is ameliorated with respect to the previous day. The specific symptoms investigated are runny nose, plugging nose, sneezing, sore throat, scratchy throat, cough, hoarseness, head congestion, chest congestion, and tiredness. Daily activities describe the ability to think clearly, have good sleep, breathe easily, exercise, work (inside and outside the home), ability to perform routine daily activities, social interactions, and personal life. The severity of the cold is evaluated as a sum of scores ranging from 0 (absent) to 7 (severe). The higher the final score, the worse the condition.

### 2.4. Measurement of Inflammatory Cytokines

The cytokines IFN-γ and IL-10 were quantified in plasma using commercially available DuoSet ELISA kits (R&D Systems Europe Ltd., distributed by Bio-Techne, Milan, Italy) according to the manufacturer’s protocols. Briefly, microtiter plates were coated overnight with monoclonal capture antibodies specific for human IFN-γ or IL-10. The following day, plates were blocked with 1% Bovine Serum Albumin (BSA) for 1 hour (h) and then incubated with plasma samples for 2 h. Subsequently, a biotinylated detection antibody was added and incubated for 2 h. Streptavidin–horseradish peroxidase conjugate was then applied, and the reaction was developed using 3,3′,5,5′-tetramethylbenzidine (Sigma-Aldrich, Saint Louis, MO, USA) as the substrate. The reaction was terminated with 2 N sulfuric acid, and absorbance was measured at 450 nm using a Tecan Spark Microplate Reader (Tecan Trading AG, Männedorf, Switzerland).

### 2.5. Statistical Analysis

The Chi-squared test for independence was used to analyze the association between categorical variables, such as the proportions of symptomatic versus asymptomatic individuals, and other dichotomous outcomes across the active and placebo groups at different time points (T1 and T2). Statistical analyses were performed using the R statistical software (version 4.3.2, R Foundation for Statistical Computing, Vienna, Austria, www.r-project.org, accessed on 12 November 2024). The results were expressed as proportions and percentages, and statistical significance was defined as *p* < 0.05.

The cytokine level significance was assessed by two-way ANOVA followed by Dunnett’s multiple comparisons test using the GraphPad Prism version 8.0.2 for Windows (GraphPad Software, San Diego, CA, USA, www.graphpad.com). The results were expressed as mean ± standard error (SEM). The statistical significance was fixed at *p* < 0.05.

## 3. Results

### 3.1. Assessment of Cold Symptoms by WURSS-21

**Figure 3:**
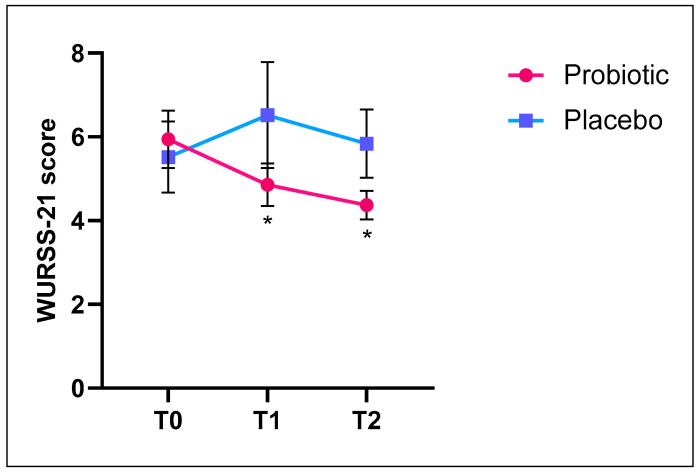
WURSS-21 questionnaire results in subjects randomized to the Probiotic and Placebo groups. The probiotic group showed a significant reduction in cold symptoms at both T1 and T2 compared to baseline values (* *p* < 0.05). Conversely, the Placebo group reported a rise in the severity of cold symptoms at T1 and T2 compared to T0.

In the WURSS-21 questionnaire, the higher the score, the worse the cold symptoms. The data showed a statistically significant decrease in the severity of cold symptoms in the Probiotic-treated group at T1 and T2 compared to T0 (intragroup variation, *p* < 0.05). In contrast, the Placebo group showed an increase in cold symptoms at T1 and T2 compared to baseline (Figure 3).

### 3.2. Assessment of the Total Number of Days with Cold Symptoms

For each group, the total number of days was calculated by multiplying the treatment duration at T1 (84 days) by the number of participants in the probiotic group (40) and the placebo group (25), resulting in 3360 days and 2100 days, respectively.

The same method was applied for the follow-up T2 (42 days), yielding 1680 days for the Probiotic group and 1050 days for the Placebo group. Subsequently, for each group, the total number of days reporting individual cold symptoms (e.g., total days with watery eyes, total days with runny nose, etc.) was calculated and expressed as a percentage. Thus, the Placebo group experienced 140 days with cold symptoms at T1, whereas the Probiotic group reported 151 days.

**Figure 4:**
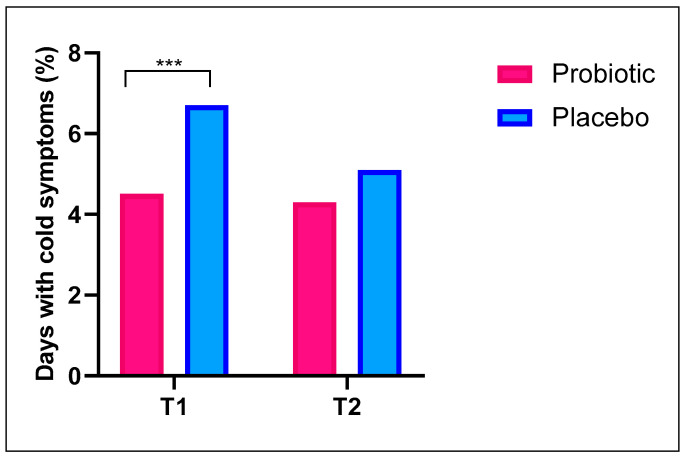
The average percentage of days in which subjects experienced cold symptoms in Probiotic and Placebo groups at T1 and T2, respectively. Chi-squared test was applied. *** *p* < 0.001.

The average duration of the illness was then calculated in subjects who contracted a cold. At T1, the average cold duration was 4.5% in the Probiotic group, compared to 6.7% in the Placebo group (*p* < 0.001). This represents a significant reduction of 32.7% in the duration of symptoms for subjects treated with probiotics compared to the Placebo group (Figure 4). At the follow-up (T2), the Probiotic group reported a total of 73 days of symptoms, whereas Placebo group reported 54 days, considering 1680 and 1050 total days under investigation, respectively (Figure 4).

### 3.3. Assessment of Typical Symptoms of Cold: Fever and Muscle Pain

For each group, the percentage of subjects reporting typical symptoms of cold (e.g., fever and muscle pain) was calculated at T1 and T2 and expressed as a percentage of the total number of subjects included in that group.

**Figure 5:**
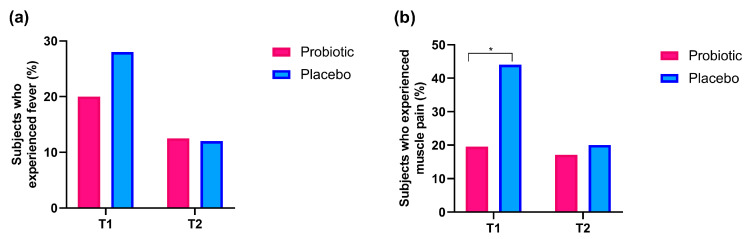
Percentage of subjects presenting fever (**a**) and muscle pain (**b**) symptoms in Probiotic and Placebo groups at T1 and T2, respectively. A chi-squared test was applied. * *p* < 0.05.

At T1, fever was present in 8 out of 40 subjects in the Probiotic group and 7 out of the 25 subjects assigned to the Placebo group, showing that 28% of the placebo-treated subjects experienced episodes of fever, compared to only 20% of the subjects supplemented with the probiotic formulation (Figure 5a). At T2, after the follow-up period, the difference between the two groups was reduced. Indeed, 5 out of 40 subjects (12.5%) in the Probiotic group and 3 out of 25 subjects (12%) in the Placebo group experienced episodes of fever (Figure 5a, not statistically significant).

Muscle pain was reported by 8 out of 40 subjects in the Probiotic group at T1 and by 11 out of 25 subjects in the Placebo group, with an incidence of 20% and 44%, respectively (*p* < 0.05, Figure 5b). Like fever, muscle pain was reported in 7 out of 40 subjects in the Probiotic group and 5 out of 25 subjects in the Placebo group at T2, with a percentage of 17.5% and 20%, respectively (Figure 5b).

### 3.4. Assessment of Subjects Using Specific Treatment for Cold and Without Any Symptoms of Cold

For each group, the percentage of subjects who needed a specific pharmacological cold treatment and the percentage who reported no symptoms at all were calculated at both T1 and T2.

**Figure 6:**
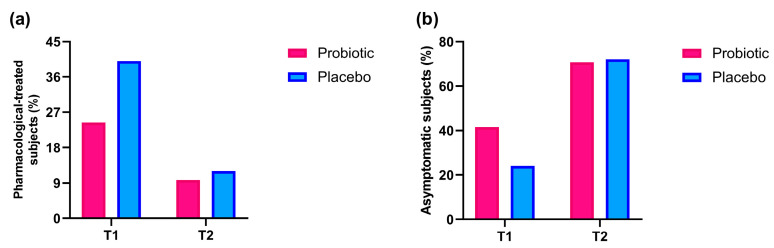
Percentage of subjects who needed treatment for cold (**a**) and without any symptom of cold (**b**) in Probiotic and Placebo groups throughout the study period.

At T1, 10 out of 40 subjects (25%) in the Probiotic group and 10 out of 25 subjects (40%) in the Placebo group required specific treatment for cold. At T2, 4 out of 40 subjects (10%) in the Probiotic group and 3 out of 25 subjects (12%) in the Placebo group used drugs to treat cold symptoms (Figure 6a). 

Furthermore, the percentage of subjects reporting no cold symptoms at T1 was higher in the Probiotic group (17 out of 40, 42.5%) compared to the Placebo group (6 out of 25, 24%), although this difference was not statistically significant. At T2, the proportion of symptom-free subjects was comparable between groups, being respectively 29 out of 40 (72.5%) in the Probiotic group and 18 out of 25 (72%) in the Placebo group (Figure 6b).

### 3.5. Cytokine Quantification

**Figure 7:**
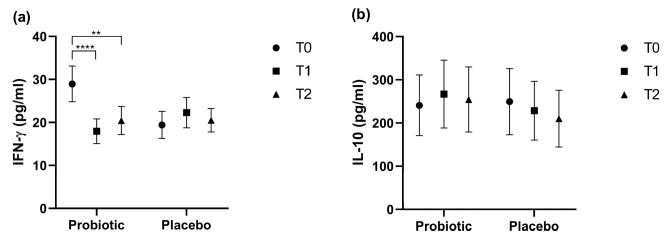
(**a**) IFN-γ and (**b**) IL-10 total cytokine dosages at each time point considered in the study. Data are reported as mean ± SEM. Two-way ANOVA followed by Dunnett’s multiple comparisons test was applied. ** *p* < 0.01; **** *p* < 0.0001.

Cytokines are signaling proteins involved in the immune response. Their quantification can provide valuable clinical information regarding the immune activation and inflammatory response of the host. In this study, circulating levels of IFN-γ, a pro-inflammatory cytokine, and IL-10, an immunomodulatory cytokine with anti-inflammatory properties, were assessed at all experimental time points (Figure 7).

The results show that IFN-γ levels significantly decreased at T1 and T2 in the Probiotic group (*p* < 0.0001 and *p* < 0.01, respectively; Figure 7a), while no significant change was observed in the Placebo group.

No statistically significant changes were recorded for IL-10 concentrations. Notably, subjects treated with probiotics had higher levels of IL-10 at T1 and T2 compared to T0, relative to the placebo group, with the most pronounced difference observed at T1(Figure 7b).

## 4. Discussion

Probiotics are beneficial live microorganisms that support health and are utilized in the treatment of various conditions, including allergic disorders and postpartum mood swings [29,30]. A systematic review indicated that probiotics may also reduce URTIs by interacting with the innate immune system [31]. Specific *Lactobacillus* species, such as *L. plantarum*, can potentially prevent influenza virus infections and enhance vaccine efficacy [32]. However, there is limited understanding of the specific strain activity, optimal dosages, and their effectiveness against various viral pathogens in healthy adults. Post-infection treatment is frequently ineffective; therefore, timing is crucial. This study evaluated the clinical efficacy of a food supplement containing probiotic strains (*L. plantarum* PBS067, *L. acidophilus* PBS066, B*. lactis* BL050) in reducing the onset of cold symptoms in healthy individuals and improving immune response. It assessed the prevalence of common cold symptoms, QoL, and levels of two emblematic proinflammatory and immunomodulatory cytokines (IFN-γ and IL-10 markers).

The WURSS-21 questionnaire indicated a significant decrease in cold symptoms among the Probiotic Group at T1 and T2 (*p* < 0.05). These results reported in Figure 3 were consistent with other studies examining the role of probiotics in preventing URTIs. In a trial conducted by Berggren et al., where participants received daily supplementation of probiotics or placebo over 12 weeks, the consumption of *Lactobacillus plantarum* HEAL 9 and *Lactobacillus paracasei* 8700:2 was associated with a significant reduction in the incidence and duration of cold episodes, as well as a decrease in the severity of pharyngeal symptoms [33]. Additionally, a follow-up study by Busch et al. demonstrated that probiotics significantly reduced the duration and severity of cold episodes [34]. The impact of a probiotic combination was also evaluated in a pediatric population. Over 3 months of daily consumption, the probiotic significantly alleviated the severity of nasal congestion/runny nose in the probiotic group compared to the placebo group [35].

The present study showed that participants treated with probiotics had a significantly shorter duration of cold symptoms than those receiving placebo, with an average cold duration of 4.5% in the Probiotic group *vs.* 6.7% in the Placebo group, corresponding to a 32.7% reduction. During the follow-up period, the days with cold symptoms were 4.2% for the Probiotic group and 5.1% for the Placebo group (Figure 4). Other studies have also reported shorter cold symptom durations in subjects treated with probiotics. A 2022 Cochrane review that analyzed 24 RCTs involving 6950 participants from children to elderly individuals across 13 countries found that probiotics could reduce the average duration of acute URTIs by approximately 1.22 days in subjects with healthy immune systems. Most of these studies administered *Lactobacillus plantarum HEAL9* and *Lactobacillus paracasei (8700:2 or N1115)* in doses of 10^9^ to 10^11^ CFU per day [31].

The effect of probiotic intake on the most prevalent cold symptoms, i.e., fever and muscle pain, was also assessed. In our study, at T1 fever was observed in 8 out of 40 individuals in the Probiotic group and 7 out of 25 individuals in the Placebo group, showing that 28% of those receiving the placebo experienced fever episodes, compared to 20% of the participants taking the probiotic formulation. At T2, following the follow-up period, the difference between the two groups narrowed. Specifically, 5 out of 40 subjects (12.5%) in the Probiotic group and 3 out of 25 subjects (12%) in the Placebo group reported episodes of fever (Figure 5a). A similar reduction in feverishness was noted in a study that examined the effects of *B. longum* BB536 *vs.* placebo in elderly individuals receiving influenza vaccinations, revealing a significant decrease in the number of individuals experiencing fever compared to the placebo group, likely due to improved innate immunity [15]. Furthermore, another study found that the daily intake of a blend of three specific probiotics for at least three months significantly reduced the severity of cold-like symptoms by almost 2 days and the number of days with fever among healthy adults [12].

Our findings also indicated a significant difference in the prevalence of muscle pain between the two groups at T1, with only 20% of participants in the Probiotic group (8 out of 40) experiencing muscle pain, compared to 44% in the Placebo group (11 out of 25). This suggests that probiotics might provide relief from muscle pain during colds. A similar beneficial effect of probiotics was also observed in an RDBPCT that examined the effects of *Bifidobacterium longum* BB536 on cold symptoms in healthy adults [36]. The results demonstrated that those taking probiotics had a significantly lower occurrence of cold symptoms and reported greater muscle pain relief than the placebo group, especially among individuals who had experienced fewer colds in the prior three years. In the present trial, the difference in muscle pain between the groups diminished at T2, similar to what was observed with fever symptoms. At this point, 17.5% of the Probiotic group (7 out of 40) and 20% of the Placebo group (5 out of 25) reported muscle pain (Figure 5b).

At T1, we showed a difference, although not significant, between the Probiotic and Placebo groups regarding the number of subjects who sought treatment for cold symptoms *vs.* those who did not (Figure 6a). Specifically, only 25% of the Probiotic group required treatment for colds *vs.* 40% of the Placebo group. By T2, the requirement for treatment decreased to 10% in the Probiotic group and 12% in the Placebo group. Moreover, 42.5% of participants in the Probiotic group reported no cold symptoms, in contrast to only 24% in the Placebo group (Figure 6b). This suggests that probiotics may effectively decrease the need for pharmacological treatment and ease cold symptoms. This conclusion is corroborated by a double-blind, randomized controlled trial conducted in a public school in central Thailand during winter, involving children aged 8 to 13 [37]. These children were randomly assigned to receive either a two-strain probiotic formulation or a placebo twice daily for three months. The study focused on the incidence of cold symptoms during that time. The results revealed that 77% of children in the probiotic group reported at least one cold symptom compared to 95% in the placebo group. As a result, those taking probiotics experienced a significantly lower risk of fever, cough, runny nose, and school absences due to the common cold. By T2, the percentage of symptom-free subjects was similar in both groups, with 72.5% in the Probiotic group and 72% in the Placebo group. This finding is consistent with earlier results on musculoskeletal pain, indicating a potential dose-dependent relationship where the effectiveness of probiotics may lessen over time after ceasing supplementation. Gut microbiota is characterized by innate stability and resilience, which makes it resistant to small perturbations (e.g., dietary changes), whereas bigger perturbations, such as antibiotic use, may disrupt this balance [38,39]. The most notable differences among all analyzed parameters between the two groups occurred at T1. By T2, these differences diminished. This change can be attributed to the end of probiotic supplementation (since the follow-up occurred six weeks after the treatment ended), and the conclusion of the flu season.

With regard to cytokine levels (Figure 7a,b), we observed that at baseline (T0), the Probiotic group showed higher IFN-γ levels compared to the Placebo group. It is important to note that participants were randomly assigned to each group, and the analysis of IFN-γ was performed only at the end of the study. Therefore, it was not possible to stratify participants based on these initial levels before intervention, and baseline IFN-γ concentrations did not influence group allocation.

At T1, the pro-inflammatory cytokine IFN-γ decreased in the Probiotic group *vs.* T0 (17.946 ± 2.857 *vs.* 28.962 ± 4.143 pg/mL, *p* < 0.0001), and increased in the Placebo group vs. T0 (22.279 ± 3.538 *vs.* 19.432 ± 3.143 pg/mL, *p* = n.s.). Although not statistically significant, the Probiotic group at T1 had higher levels of IL-10 *vs.* T0 (266.98 ± 78.432 *vs.* 240.967 ± 70.238 pg/mL, *p* = n.s.). Probiotics may play a role in modulating the immune response during viral and bacterial infections by influencing cytokine levels [40]. Lactic acid bacteria (LAB) provide beneficial effects through their interactions with host intestinal epithelial cells. *Lactobacilli* and *Bifidobacteria* are members of this group, predominantly resident in the intestinal tract, where they enhance the host’s immune response against viral infections. A study by Kanmani et al. determined whether in vitro exposure to selected LAB (isolated from traditional Korean fermented foods) beneficially modulates innate antiviral immune responses in human colon monocytic cells (HCT116 cells and THP-1). The study revealed that LAB enhanced IL-10 levels in THP-1 cells and promoted IFN-β production in HCT116 cells when exposed to PolyI:C, a synthetic double-stranded RNA analog that triggers an inflammatory response similar to that evoked by viral double-stranded RNA. LAB also offers cellular protection by preserving tight junction proteins, such as zonula occludens-1 and occludin. The positive effects of these LAB were attributed to their ability to modulate the interferon regulatory factor 3 (IRF3) and nuclear factor-kappa B (NF-κB) pathways [40]. Similarly to what occurred in our study, the effect of probiotics in reducing levels of the pro-inflammatory cytokine IFN-γ in the blood while simultaneously increasing levels of the anti-inflammatory cytokine IL-10 was also reported by Altadill et al. in participants who received a probiotic supplementation over 12 weeks [41]. The host’s inflammatory status influences the level of circulating cytokines. It is reasonable to assume that individuals who have recently experienced URTIs may have heightened baseline levels of cytokines in their local and systemic environments, which could necessitate extended treatment periods with probiotics to stabilize cytokine levels [41].

The precise molecular mechanisms by which probiotics prevent colds are not yet completely understood. Probiotics, especially *Lactobacilli*, can enhance resistance to bacterial URTIs and modulate immune responses through various components, including peptidoglycans and metabolites (i.e., SCFAs and bacteriocins). The most likely ways through which oral administration of *Lactobacilli* can enhance immune function include (i) *direct immune cell migration*: probiotics stimulate immune cells in the intestine, which then travel to the lungs, thereby strengthening defense against infections like pneumonia and influenza; (ii) *metabolites*, such as SCFAs produced by lactobacilli*,* positively influence lung immunity by increasing the production of immune cells and fostering anti-inflammatory responses; (iii) *gut–respiratory microbiota interaction*: there is a notable relationship between gut microbiota and airway immunity, where metabolites from the gut may help modulate respiratory immune responses, suggesting potential therapeutic avenues for respiratory diseases [42].

Vitamin C is directly involved in immune function by strengthening the skin’s barrier, supporting the function of white blood cells, and protecting immune cells from oxidative stress. Although vitamin C can prevent and treat respiratory and systemic infections [43,44], we chose not to include it in the probiotic formulation due to its potential impact on the long-term stability of the probiotic strains. Our primary goal was to assess the effects of probiotics on cold symptoms, not their synergistic effect with vitamin C.

The limitations of our study include reliance on a questionnaire to self-report symptoms for the diagnosis of the common cold. Furthermore, factors such as dose, duration of administration, diet, and seasonality may impact effectiveness. Participants were instructed to avoid dietary supplements containing probiotics and to limit the consumption of probiotic-rich foods, such as yogurt and fermented products. However, a comprehensive analysis of the nutritional components present in the diet was not performed, and this will be considered in subsequent studies. Further research is required to entangle the probiotic mechanism of action in alleviating cold symptoms in healthy individuals.

## 5. Conclusions

In conclusion, the probiotic combination consisting of *Lactiplantibacillus plantarum* PBS067, *Lactobacillus acidophilus* PBS066, and *Bifidobacterium animalis* subsp. *lactis* BL050, demonstrated the ability to alleviate cold symptoms and decrease levels of pro-inflammatory cytokines. This probiotic formulation may modulate the immune response, suggesting a possible anti-inflammatory effect through multiple pathways. Further trials with a larger sample size of patients will be needed to corroborate the data provided herein.

## Data Availability

The data presented in this study are available on request from the corresponding author.

## Supplementary Materials

The following supporting information can be downloaded at: https://www.mdpi.com/article/10.3390/nu17091490/s1, Table S1: Inclusion and exclusion criteria applied to subjects of both sexes aged between 18 and 44 years enrolled in the study.

## Abbreviations

The following abbreviations are used in this manuscript:

CFU	Colony-forming unit
LAB	Lactic acid bacteria
QoL	Quality of life
RCT	Randomized clinical trial
RDBPCT	Randomized, double-blind, placebo-controlled trial
SCFAs	Short-chain fatty acids
URTIs	Upper respiratory tract infections
