# Supplementary material for: Clinical Efficacy of Probiotics for Relieving Cold Symptoms in Healthy Individuals: A Randomized, Double-Blind, Placebo-Controlled Clinical Trial
Source: Nutrients. 2025 Apr 28;17(9):1490. doi: 10.3390/nu17091490 (PMC12073269; doi:10.3390/nu17091490)
Supplement: Supplementary file 1 [file nutrients-17-01490-s001.zip › nutrients-3540668-supplementary.pdf]

## Supplementary data

**Supplementary Table S1. Inclusion and exclusion criteria applied to subjects of both sexes aged between 18 and 44 years enrolled in the study.**

| |
|---|
| **Inclusion criteria were:** |
| Willingness and ability to participate in the study |
| Willingness not to change their normal daily routine (lifestyle, physical activities, etc.) throughout the duration of the study |
| Willingness not to change their normal diet for the entire duration of the study |
| Willingness to use only the product to be tested for the entire duration of the study |
| Willingness not to use similar products for the entire duration of the study |
| Willingness not to use products that may interfere with the product to be tested |
| Willingness not to participate in similar studies |
| Signed the informed consent and be aware of the procedures of the study. |
| **Exclusion criteria were:** |
| Subjects who did not respect the inclusion criteria |
| Subjects with suspected or confirmed sensitivity to one or more components of the product |
| Subjects with a clear history of chronic diseases (congenital cardiovascular diseases, diseases of the liver and kidney, or immunodeficiency) |
| Subjects undergoing pharmacological and/or antibiotic treatment (currently) |
| Subjects who had other ongoing concomitant pathologies (immune, infectious, respiratory, or gastro-intestinal) |
| Subjects undergoing treatment to modulate the immune system in the last 4 weeks |

| |
|---|
| Subjects recently (less than 3 months) subjected to immunosuppressant therapy |
| Subjects with ongoing serious illnesses |
| Subjects using illicit drugs and/or alcohol abuse |
| Subjects who were deemed by the investigator to be unsuitable for participation for any reason |
| Subjects who were unable to communicate or cooperate with the physicians involved in the trial due to speech problems, mental retardation, or impaired brain function. |
